# Transplante Cardíaco Ortotópico em Receptor com Covid-19

**DOI:** 10.36660/abc.20220234

**Published:** 2023-03-20

**Authors:** Leonardo Rufino Garcia, André Monti Garzesi, Julia Bazzo Sinatora, Rejane Maria Tommasini Grotto, Andréia Cristina Passaroni, Nelson Leonardo Kerdahi Leite de Campos, Antônio Sérgio Martins, Marcello Laneza Felicio, Flávio de Souza Brito

**Affiliations:** 1 Hospital das Clínicas de Botucatu UNESP Botucatu SP Brasil Serviço de Cirurgia Cardiovascular e Transplante Cardíaco do Hospital das Clínicas de Botucatu – UNESP, Botucatu, SP – Brasil; 2 Laboratório de Biotecnologia Aplicada Hospital das Clínicas de Botucatu UNESP Botucatu SP Brasil Laboratório de Biotecnologia Aplicada do Hospital das Clínicas de Botucatu – UNESP, Botucatu, SP – Brasil

**Keywords:** SARS-CoV2, Pandemias/prevenção e controle, Betacoronavirus/complicações, Transplante de Coração, Imunossupressão, Comorbidade, Idoso, Linfopenia, Inflamação

## Introdução

A pandemia pelo coronavírus acometeu mais de 480 milhões de pessoas ao redor do mundo.^1^ Receptores de órgãos sólidos constituem um grupo altamente vulnerável devido à necessidade de imunossupressão e às várias comorbidades que podem estar associadas,^2^ muitas das quais persistem mesmo após a realização do transplante.^3^

Pacientes transplantados podem apresentar um espectro de manifestações clínicas decorrentes da COVID-19, desde assintomáticos, sintomas leves ou falência respiratória aguda e óbito.^4^ Alguns estudos sugerem que no caso de quadros com desfecho favorável, a imunização prévia e a imunossupressão necessária após transplante contribuem para uma resposta inflamatória menos exacerbada e, consequentemente, menor lesão orgânica.^3,5^ Entretanto, alguns relatos e revisões da literatura evidenciam pior prognóstico em transplantados, possivelmente devido às comorbidades, idade avançada e linfopenia frequentemente presentes.^3,4,6^

Dessa forma, descrevemos o caso de paciente com RT-PCR positivo para Sars-Cov-2 submetido a transplante cardíaco ortotópico bicaval com evolução satisfatória e sem sequelas respiratórias crônicas até o momento. Trata-se de um dos primeiros casos descritos submetido a transplante de coração durante vigência de COVID-19.

## Descrição

Homem, 64 anos, com insuficiência cardíaca de etiologia isquêmica com fração de ejeção reduzida em classe funcional IV. Hipertenso, diabético, submetido à cirurgia de revascularização do miocárdio em 2014 e implante de cardiodesfibrilador em 2019. Havia recebido duas doses de vacina contra COVID-19, do fabricante Astrazeneca, completando o esquema em setembro de 2021. Internado em hospital terciário, foi priorizado para transplante cardíaco em uso de dobutamina e assistência circulatória com balão intra-aórtico e classificado como INTERMACS-3.^7^ Estava em ventilação espontânea, sem queixas respiratórias e sem necessidade de oxigenioterapia. Foi submetido a transplante cardíaco ortotópico bicaval em fevereiro de 2022 sem intercorrências cirúrgicas com tempo de isquemia total de quatro horas e 30 minutos.

No primeiro dia pós-operatório, o exame de RT-PCR para COVID-19 coletado imediatamente antes da cirurgia de acordo com protocolo institucional resultou positivo. A variante viral identificada posteriormente pelo método de sequenciamento em larga escala foi a Ômicron BA.1. Foi iniciada imunossupressão habitual com tacrolimus, micofenolato mofetil e corticosteróides. Sulfametoxazol/trimetoprima e ganciclovir foram introduzidos para profilaxias de infecções em imunossuprimidos. Para profilaxia cirúrgica foram utilizados meropenem e vancomicina por cinco dias. Seguiu-se evolução favorável com diminuição gradual de drogas vasoativas e do suporte de balão intra-aórtico até sua retirada. O paciente foi mantido em isolamento, em ventilação espontânea, em uso de cateter nasal de oxigênio a 2l/min e sem queixas respiratórias. Realizada tomografia de tórax no 12º dia de pós-operatório, com achados de aspecto atípico para pneumonia de etiologia viral. Novo RT-PCR colhido no 13º dia resultou negativo. Recebeu alta hospitalar 30 dias após o transplante.

Durante seguimento ambulatorial três meses após o transplante houve elevação de marcadores laboratoriais de rejeição ao enxerto (troponina, peptídeo natriurético atrial e proteína C reativa), porém com ausência de sintomas. Optou-se por realização de pulsoterapia com metilprednisolona. Ecocardiograma sem alterações em comparação a exame prévio, mantinha fração de ejeção de 60% e sem aumento de espessura das paredes. Biópsia endomiocárdica mostrou fragmento com infiltrado inflamatório predominantemente linfocitário perivascular e ausência de agressão ao cardiomiócito, sendo classificação ISHLT (International Society for Heart and Lung Transplantation) para rejeição celular grau zero e para rejeição humoral pAMR zero.^8^ Mantida imunossupressão com tacrolimus, prednisona e micofenolato mofetil, paciente evoluiu com normalização dos marcadores laboratoriais de rejeição e permaneceu assintomático.

## Discussão

A necessidade de imunossupressão crônica em pacientes transplantados é fator de risco para complicações e desfecho negativo quando associada a infecções. Devido a experiência da pandemia por H1N1 em 2009, esperava-se que tais pacientes tivessem pior prognóstico quando acometidos por COVID-19, evoluindo com pneumonia e síndrome do desconforto respiratório agudo mais frequentemente do que a população geral. Porém, uma hipótese é que a imunossupressão nesses casos reduza a intensidade da síndrome hiperinflamatória secundária à tempestade de citocinas, presente na maioria dos casos de morte por coronavírus.^5^

Apesar de manifestações clínicas semelhantes à população geral, a mortalidade parece ser maior no grupo de pacientes transplantados cardíacos conforme demonstrado em alguns relatos de caso.^9-11^ Contudo, não é possível dizer se a causa de pior prognóstico está relacionada à imunossupressão ou às múltiplas comorbidades, idade avançada ou quadros infecciosos mais graves em tais pacientes.^4^ Há relatos de piores desfechos com infecção por COVID-19 na população geral relacionados com linfopenia,^6^ que também pode estar presente nos pacientes transplantados devido a efeito colateral de medicações imunosupressoras. Além disso, o prognóstico também se mostrou pior quando há elevação de biomarcadores como proteína C reativa e procalcitonina, tanto na população geral quanto nos pacientes transplantados.^12,13^

O manejo dos imunossupressores nesses casos deve ser individualizado, pesando-se o risco de piora infecciosa e o risco de rejeição ao enxerto. Ballout et al.,^4^ elaboraram um fluxograma para guiar a imunossupressão em pacientes transplantados cardíacos que apresentaram COVID-19: manutenção de micofenolato mofetil em doses mais baixas caso o paciente não apresentasse linfopenia ou sinais de infecção grave; inibidores da calcineurina foram mantidos em faixa terapêutica e prednisona também foi utilizada, exceto quando houvesse indicação do uso de dexametasona (nesses casos, a prednisona foi suspensa durante tratamento com dexametasona). Além disso, se o paciente apresentasse elevação de marcadores de rejeição e enzimas cardíacas, recomendou-se a realização de biópsia endomiocárdica a fim do diagnóstico diferencial entre rejeição ao enxerto e miocardite viral por COVID-19.^4^

Em revisão da literatura, alguns autores concluíram que a resposta imune inata nos pacientes transplantados cardíacos deva ser parecida à da população geral, com níveis semelhantes de marcadores inflamatórios e interleucina-6 em ambos os grupos de pacientes hospitalizados. Em relação à resposta humoral, também se notou que a formação de anticorpos foi semelhante entre os pacientes transplantados e os não transplantados, sendo que anticorpos específicos foram identificados após uma a duas semanas do início dos sintomas e permaneceram por um período médio de dois meses, podendo chegar até seis meses.^14^

Em outro estudo com 232 pacientes concluiu-se que a formação de anticorpos contra Sars-Cov-2 em imunossuprimidos foi semelhante ao que se observa na população geral, bem como a taxa de infecção entre os dois grupos. Entretanto, neste estudo, pacientes transplantados evoluíram com pior desfecho, possivelmente devido à presença de múltiplas comorbidades associadas.^15^

Outro fator que pode ter contribuído para o controle da resposta inflamatória e evolução favorável foi a completude do esquema vacinal à época com uma vacina previamente testada e com eficácia comprovada. Como já mencionado, a despeito da imunossupressão, pacientes transplantados cardíacos têm resposta imune semelhante à população geral quando infectados pelo coronavírus.^15^ Dessa forma, mesmo com a manutenção da imunossupressão, o paciente em questão provavelmente ainda mantinha concentrações de anticorpos específicos, o que pode ter contribuído para o desfecho satisfatório. No caso exposto, o principal diferencial consistiu na vacinação prévia do paciente, o que não foi relatado nos estudos descritos pois as várias vacinas hoje disponíveis ainda se encontravam em fase de desenvolvimento.

Outro ponto ainda a ser elucidado em outros estudos e amostras maiores de pacientes consiste em entendermos como o Sars-Cov-2 pode afetar a função do enxerto e eventualmente causar rejeição uma vez que já é amplamente descrito que o vírus possui tropismo por células cardíacas podendo causar injúria miocárdica e miocardite.

## Conclusão

Descrevemos um caso de paciente submetido a transplante cardíaco em vigência de COVID-19 e previamente vacinado, com evolução satisfatória. O manejo da imunossupressão deve ser considerado de forma individual nesse cenário. Para a tomada de decisões clínicas, o risco de rejeição do enxerto com piora da doença que indicou o transplante deve ser levado em conta frente à gravidade da infecção quando associada à inflamação sistêmica exacerbada.
