# Supplementary material for: Transplante Cardíaco Ortotópico em Receptor com Covid-19
Source: Arq Bras Cardiol. 2023 Mar 20;120(3):e20220234. [Article in Portuguese] doi: 10.36660/abc.20220234 (PMC10392859; doi:10.36660/abc.20220234)
Supplement: Translation

# Translation: Orthotopic Heart Transplantation in a Covid-19 Recipient

**Authors:** Leonardo Rufino Garcia, André Monti Garzesi, Julia Bazzo Sinatora, Rejane Maria Tommasini Grotto, Andréia Cristina Passaroni, Nelson Leonardo Kerdahi Leite de Campos, Antônio Sérgio Martins, Marcello Laneza Felicio, Flávio de Souza Brito

**Affiliations:** 1 Brazil Serviço de Cirurgia Cardiovascular e Transplante Cardíaco do Hospital das Clínicas de Botucvvatu – UNESP, Botucatu, SP – Brazil; 2 Brazil Laboratório de Biotecnologia Aplicada do Hospital das Clínicas de Botucatu – UNESP, Botucatu, SP – Brazil

**Keywords:** SARS-CoV2, Betacoronavirus/complications, Heart Transplantation, Imunosuppresion, Comorbidity, Aged, Limphopenia

## Introduction

The coronavirus pandemic has affected more than 480 million people around the world.^1^ Solid organ recipients constitute a highly vulnerable group due to the need for immunosuppression and the various comorbidities that may be associated,^2^ many of which persist even after transplantation.^3^

Transplanted patients can present a spectrum of clinical manifestations resulting from COVID-19: from asymptomatic, mild symptoms or acute respiratory failure and death.^4^ Some studies suggest that in the case of conditions with a favorable outcome, previous immunization and irreplaceable post-transplantation immunosuppression contribute to a response of less exacerbated inflammation and, consequently, less organic damage.^3,5^ Nonetheless, some reports and literature reviews show worse prognosis in transplanted patients, possibly due to comorbidities, advanced age, and lymphopenia often present.^3,4,6^

Indeed, we describe the case of a patient with positive RT-PCR for Sars-Cov-2 who underwent bicaval orthotopic heart transplantation with satisfactory evolution and no chronic respiratory sequelae so far. This is one of the first reported cases submitted to heart transplantation concomitant with COVID-19.

## Description

Male, 64 years old, heart failure with a reduced ejection fraction of ischemic etiology in NYHA (New York Heart Association functional classification) IV. Hypertensive and diabetic, he was submitted to myocardial revascularization in 2014 and cardioverter-defibrillator implantation in 2019. He had received two doses of the AstraZeneca vaccine against COVID-19, with the last dose in September 2021. Admitted to a tertiary hospital, was prioritized for cardiac transplantation using dobutamine and circulatory assistance with an intra-aortic balloon pump and classified as INTERMACS-3.^7^ He was on spontaneous ventilation, with no respiratory complaints and no need for oxygen therapy. He underwent an uneventful bicaval orthotopic heart transplantation in February 2022 with a total ischemic time of four hours and 30 minutes.

On the first postoperative day, an RT-PCR exam for COVID-19 done immediately before surgery according to the institutional protocol was positive. The viral variant identified later by the large-scale sequencing method was an Omicron BA.1. Usual immunosuppression with tacrolimus, mycophenolate, and corticosteroids had then started. Trimethoprim/sulfamethoxazole and ganciclovir were introduced according to infective prophylaxis in immunosuppressed patients. For surgical prophylaxis, meropenem and vancomycin were used for five days. A favorable evolution followed with a gradual decrease in the vasoactive agent’s dosage and intra-aortic balloon support until its removal. The patient was kept in respiratory isolation, in spontaneous ventilation, using a nasal oxygen catheter at 2l/min, and without respiratory complaints. Chest tomography on the 12th postoperative day revealed an atypical appearance of viral pneumonia. A new RT-PCR performed on the 13th day was negative. He was discharged 30 days after the transplant.

During outpatient follow-up, three months after transplantation, there was an increase in laboratory markers of graft rejection (troponin, atrial natriuretic peptide and C-reactive protein), but symptoms were absent. The medical staff then opted for pulse therapy with methylprednisolone. The echocardiogram showed no changes compared to the previous examination, with an ejection fraction of 60% and no increase in wall thickness. Endomyocardial biopsy tissue showed a fragment with predominantly perivascular lymphocytic inflammatory infiltrate and absence of aggression to the cardiomyocyte, with ISHLT classification (International Society for Heart and Lung Transplantation) grade zero for cellular rejection and pAMR zero for humoral rejection.^8^ Immunosuppression was maintained with tacrolimus, prednisone, and mycophenolate. The patient evolved with the normalization of laboratory markers of rejection and remained asymptomatic.

## Discussion

The need for chronic immunosuppression in transplant patients is a risk fator for complications and negative outcomes when associated with infections. Due to the experience of the H1N1 pandemic in 2009, the medical community expected that such patients would have a worse prognosis when affected by COVID-19, progressing to pneumonia and respiratory distress syndrome more frequently than the general population. However, one hypothesis is that the immunosuppression in these cases reduces the intensity of the secondary hyperinflammatory syndrome due to the cytokine storm present in most cases of death from COVID-19.^5^

Despite similar clinical manifestations to the general population, mortality seems to be higher in the group of heart transplant patients, as demonstrated in some case reports.^9-11^ However, it is not possible to say whether the cause of the worse prognosis is related to immunosuppression itself or multiple comorbidities, advanced age, or more severe infective scenarios in such patients.^4^ There are reports of worse outcomes with COVID-19 infection in the general population related to lymphopenia,^6^ which may also be present in transplanted patients due to the side effect of some immunosuppressive medications. In addition, the prognosis was also worse when there had been an increase in biomarkers such as C reactive protein and procalcitonin, both in the general population and transplanted patients.^12,13^

The management of immunosuppressants in these cases must be individualized, weighing the risk of infection worsening and graft rejection. Ballout et al.^4^ developed a flowchart to guide immunosuppression in heart transplant patients who presented with COVID-19: maintenance of mycophenolate at lower doses if the patient does not have lymphopenia or signs of severe infection; calcineurin inhibitors were kept in the therapeutic range, and prednisone was also used, except when there was an indication for the use of dexamethasone (in these cases, prednisone was suspended during treatment with dexamethasone). Furthermore, if the patient presented an elevation of rejection markers and cardiac enzymes, an endomyocardial biopsy was recommended to diagnose the difference between graft rejection and viral myocarditis due to COVID-19.^4^

In a review of the literature, some authors concluded that the innate immune response in heart transplant patients seems to be similar to the general population, with similar levels of inflammatory markers and interleukin-6 in both groups of hospitalized patients. Regarding the humoral response, it was also noted that the formation of antibodies was similar between transplanted and non-transplanted patients, with specific antibodies identified after one to two weeks of symptom onset, and they stayed for an average period of two months and could reach up to six months.^14^

Another study with 232 patients concluded that the formation of antibodies against Sars-Cov-2 and the infection rate in immunosuppressed individuals were similar to what is observed in the general population. However, in this study, transplanted patients evolved with a worse outcome, possibly due to multiple associated comorbidities.^15^

Another factor that may have contributed to the control of the inflammatory response and favorable evolution was completing the vaccination schedule at that time with a previously tested and proven effective vaccine. As already mentioned, despite immunosuppression, heart transplant patients have a similar immune response compared to the general population when infected by the Sars-Cov-2.^15^ Thus, even with the maintenance of immunosuppression, the patient in question probably maintained blood concentrations of specific antibodies, which may have contributed to the satisfactory outcome. In this case, the main differential consisted of the previous patient vaccination, which was not reported in the studies described since the various vaccines available today were still in development at that time.

Another point still to be elucidated in other studies and larger samples of patients is understanding how Sars-Cov-2 can affect graft function and eventually cause rejection since it is already widely described that the virus has cardiac cell tropism and may cause myocardial injury and myocarditis.

## Conclusion

We describe the case of a patient who underwent heart transplantation during active COVID-19 but was previously vaccinated, with favorable evolution. The management of immunosuppression must be considered individually in this scenario. In the clinical decision-making process, the risk of graft rejection with worsening of the primary disease that indicated the transplant must be considered against the severity of the coronavirus infection when associated with exacerbated systemic inflammation.
